# Changes in Generations of PAMAM Dendrimers and Compositions of Nucleic Acid Nanoparticles Govern Delivery and Immune Recognition

**DOI:** 10.1021/acsbiomaterials.5c00336

**Published:** 2025-05-20

**Authors:** Yelixza I. Avila, Laura P. Rebolledo, Nathalia Leal Santos, Brandon Rawlins, Yasmine Radwan, Melanie Andrade-Muñoz, Elizabeth Skelly, Morgan R. Chandler, Luciana N. S. Andrade, Tae Jin Kim, Marina A. Dobrovolskaia, Kirill A. Afonin

**Affiliations:** † Department of Chemistry, 14727 University of North Carolina at Charlotte, Charlotte, North Carolina 28223, United States; ‡ Center for Translational Research in Oncology (LIM24), Instituto do Câncer do Estado de São Paulo, Hospital das Clínicas da Faculdade de Medicina da Universidade de São Paulo, Comprehensive Center for Precision Oncology, Universidade de São Paulo, São Paulo 01246-000, Brazil; § MIMETAS US, Inc, Gaithersburg, Maryland 20878, United States; ∥ Department of Physical Sciences, 552947 West Virginia University Institute of Technology, Beckley, West Virginia 25801, United States; ⊥ Nanotechnology Characterization Laboratory, Cancer Research Technology Program, Frederick National Laboratory for Cancer Research, Frederick, Maryland 21702, United States

**Keywords:** NANPs, PAMAM dendrimers, delivery, immune response, interferons, spheroids

## Abstract

Nucleic acid nanoparticles (NANPs) are promising immune modulators due to their well-established structural properties and distinct structure–activity relationship with the immune system. We previously identified that NANPs’ size, shape, composition, and type of delivery vehicle define their uptake by immune cells and subsequently induced cytokine profile. In this work, we examined the delivery efficiencies and immunological impacts of two representative NANPs—DNA cubes and RNA cubes—complexed with a benchmark delivery vehicle, Lipofectamine 2000 vs. different generations of amine-terminated poly­(amidoamine) dendrimers. Using molecular dynamics simulations, we modeled dendrimer interactions with nucleic acid cargos. Next, we used traditional 2D and more recently established 3D cell cultures to assess dendrimers’ influence on NANPs uptake. Immune activation was evaluated in several cell lines engineered with reporter genes driven by key immune signaling pathways. Specifically, HEK-lucia reporter cells were used to evaluate RIG-I activation, while THP1-Dual cells provided quantitative readouts for both IRF and NF-κB transcription factor activity. Our findings demonstrate that both dendrimer generation and NANP composition influence cellular uptake and immune responses. This study underscores the importance of formulation in shaping NANPs’ biological properties and further advances the understanding of their immunological properties critical for the development of NANPs-based adjuvants.

## Graphical abstract

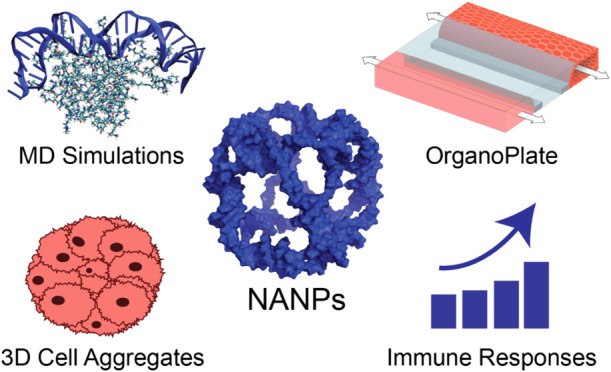

## Introduction

DNA and RNA are biopolymers with unique structural components that define their diverse functions and interactions with other molecules; even minor structural differences in these biopolymers impact their physicochemical properties and therapeutic outcomes. Since naked and carrier-free nucleic acids have limited systemic delivery, expanding the range of delivery vehicles is crucial for the field of nanomedicine. Effective carriers must enhance cellular uptake, protect cargo from degradation, and minimize immune recognition to ensure therapeutic efficacy.^1–6^ Shielding nucleic acids from interactions with specific pattern recognition receptors to avoid immune detection also reduces unwanted responses, improving the safety of NANPs-based formulations. Ultimately, selecting the right delivery approach is critical for optimizing nanotherapeutic efficacy and the success of new drug delivery platforms.

Poly­(amidoamine) (PAMAM) dendrimers are synthetic polymeric nanoparticles with chemical modifications to facilitate interactions with therapeutic nucleic acids.^7^ Amine-terminated PAMAM dendrimers are synthesized with positively charged surface groups that can electrostatically bind to nucleic acid nanoparticles (NANPs),^8^ a new generation of rationally designed nucleic acids suitable for broad biomedical applications.^4,9–17^ Dendrimers, as polymeric nanoparticles, are synthesized from ethylene diamine or diamino benzidine core by subsequent addition of branches, resulting in distinct generations from generation (G) 0 to 10 with G3 through G5 most commonly explored for drug delivery.^18^ Among these generations, G0 is the dendrimer’s core, which serves as the starting point. With each subsequent synthesis step, the dendrimer branches out from its surface terminal groups, approximately doubling in both molecular weight and number of terminal surface groups with each generation.^19^ While higher dendrimer generations have a greater number of terminal surface groups, the higher density of these groups limits their availability for interaction with drugs or nucleic acids, explaining the popularity of G3-G5 for drug delivery.^20–24^ The original studies by Tomalia et al. demonstrated no apparent advantage of using higher-generation PAMAM dendrimers for gene delivery.^25^ Numerous studies by the National Cancer Institute Nanotechnology Characterization Lab and other researchers have clearly demonstrated that the biological and immunological properties of dendrimers are largely determined by nature (amine- vs. – hydroxy- vs. carboxy-terminal groups) and density of terminal surface groups, with higher generation cationic (amine- or guanidine-terminated) dendrimers being more reactive.^19,25–32^ These properties were confirmed across other types of dendrimers, including triazine dendrimers.^28,33,34^ Some recent studies suggested that some generations may provide a greater capability of binding more than one therapeutic payload^24,35–38^ and that higher dendrimer generations are protected from immune recognition.^39^

Understanding NANP-dendrimer interactions is critical, as immunological profiling has emerged as a key approach in advancing therapeutic nucleic acids and NANP technologies. In previous studies, we developed standardized protocols to assess the pro-inflammatory properties of NANPs in human peripheral blood mononuclear cells (PBMCs)^5^ and confirmed their reproducibility across more than 60 different NANPs tested by multiple laboratories in PBMCs collected from healthy human donors. These studies revealed that, unlike traditional therapeutic nucleic acids, NANPs without delivery carriers are immunoquiescent, and plasmacytoid dendritic cells serve as primary interferon (IFN) producers in PBMCs transfected with Lipofectamine 2000 (L2K)-complexed NANPs.^5,40,41^ We also demonstrated that NANP immunostimulation depends on their dimensionality, composition, and functionalization, with type I IFNs being key biomarkers of NANP internalization by phagocytes. Through our work with PBMCs and other immune cells, including microglia cultures, we further demonstrated the utility of reporter cell lines in gaining mechanistic insights into NANP-mediated immunostimulation via various innate immune pattern recognition receptors.^14,42–44^

In this work, we explored several generations of PAMAM dendrimers as delivery vehicles for NANPs (Figure 1). We selected two representative NANPs based on the prior studies: RNA cubes were used as a lead candidate due to their consistently high immunostimulatory activity, while DNA cubes were used as an internal control because they exhibited minimal immunostimulation.^5,41,44^ We investigated how dendrimer generation affects the delivery efficiency of these NANPs in traditional monolayer cultures, as well as in 3D spheroid models and innovative OrganoPlate models that replicate tissue architecture and cellular interactions. L2K was chosen as the control carrier for all cell studies due to its compatibility with various NANPs and other nucleic acid cargos and its established baseline data with optimized conditions from prior works.

**Figure 1:**
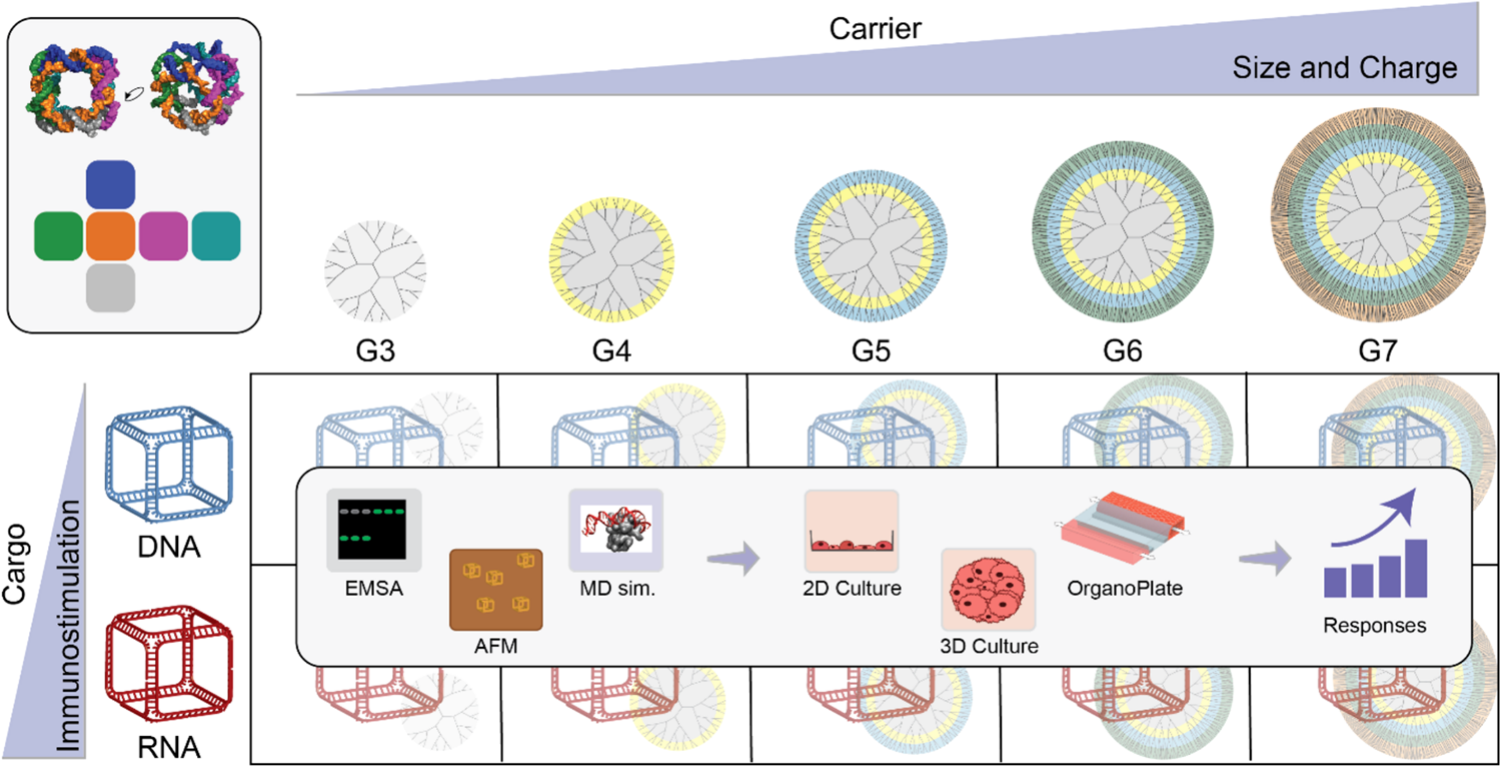
Graphical summary of the current study. Representative NANPs are complexed with PAMAM dendrimers of increasing generation, which are then characterized by both their physicochemical properties and *in vitro* behavior.

We supported our experimental work with extensive molecular dynamics (MD) simulations to better understand the structures of our formulations. The dynamics of PAMAM and poly­(propyleneimine) (PPI) dendrimers have been extensively studied through MD simulations using various force fields, including Dreiding, CVFF, AMBER, GAFF, CHARMM, GROMOS, and GROMACS.^45–51^ MD simulations have explored the effect of pH on the complexation between small PAMAM dendrimers (G0 and G1) and a 21-base-pair siRNA over 20 ns,^52^ as well as the binding enthalpy between the siRNAs and G7 PAMAM dendrimers using molecular mechanics Poisson–Boltzmann surface area (MM-PBSA) calculations based on a 10 ns MD simulation.^53^ Beyond siRNAs, MD simulations have also examined the interactions between small dendrimers and RNA or single-stranded DNA.^54–56^ Building on prior studies, we performed 100 ns MD simulations to characterize the complexation between amine-terminated PAMAM dendrimers (G3–G7) and representative RNA or DNA duplexes of identical sequence and length.

## Materials and Methods

### Molecular Dynamics Simulations

The conformation of protonated amine-terminated PAMAM dendrimers using 20–25 ns molecular dynamics (MD) simulations has previously been investigated.^58^ Their simulations were performed using the general AMBER force field (GAFF) under neutral salt (0 M) and neutral pH conditions. For this study, equilibrated G3-G7 PAMAM dendrimers were obtained from their database, and the most recent AMBER GAFF was applied.^48^ The initial PAMAM dendrimers were prepared at neutral pH, ensuring that all primary amines were protonated. Each dendrimer was solvated with TIP3P water using AMBER TIP3P force fields, and protonated amines were neutralized with Cl^–^ ions. Additional K^+^ and Cl^–^ ions were included to achieve a final salt concentration of 0.052 M. Ionization parameters were defined using AMBER ion force fields (frcmod.ions1lm_126_tip3p).^59–61^ To prepare the system for MD simulations, the solvated structure was initially minimized while constraining the PAMAM dendrimer atoms. This was followed by equilibration using a 200 ps simulation at 310.15 K with Langevin dynamics, maintaining constraints on all dendrimer atoms. Subsequently, an unconstrained minimization was performed for 20,000 steps, followed by an equilibration step lasting 500 ps under constant pressure (1 atm) and temperature (301.15 K). The production of MD simulations were carried out under NPT conditions, maintaining pressure at 1 atm using the Langevin piston method^62^ and temperature at 310.15 K with weakly coupled Langevin dynamics. The system was simulated using periodic boundary conditions, employing the particle mesh Ewald method^63^ for full electrostatics calculations. Short-range nonbonded interactions were evaluated at each step using a 12 Å cutoff with a smooth switching function for van der Waals interactions. The production simulations were conducted for 60 ns with a 2 fs time step, utilizing the NAMD simulation package.

The RNA duplex used in this study corresponded to the same Dicer substrate RNA sequence previously investigated for interactions with cationic delivery vehicles, including bolaamphiphiles.^15,64–66^ The DNA duplex shared the same sequence but with uracil replaced by thymine and constructed as a B-form helix (SI Figure S1). The initial structures of both DNA and RNA duplexes were generated using Discovery Studio, with AMBER DNA.OL15 force fields applied to DNA and AMBER RNA.OL3 force fields applied to RNA. Solvation and ionization conditions were identical to those used for PAMAM dendrimers. The same MD simulation protocols were applied to both nucleic acid systems, and production simulations were performed for 50 ns with a 2 fs time step. Following the completion of MD simulations for PAMAM dendrimers and nucleic acids, water molecules and ions were removed from the final MD snapshot of each system. To analyze complexation, nucleic acids and PAMAM dendrimers were initially positioned 5 Å apart before applying solvation and ionization conditions identical to those described above. The same MD simulation protocols were used for all eight systems, which included DNA and RNA complexed with G3, G4, G5, G6, and G7 dendrimers. The production simulations were conducted for 100 ns, with the final 20 ns of MD trajectories used for data analysis. Hydrogen bond (HB) interactions, solvent-accessible surface area, and the population of protonated amine groups near phosphate groups were calculated using the CPPTRAJ module in the AMBER MD simulation package. Binding free energies of nucleic acids to PAMAM dendrimers were computed using molecular mechanics Poisson–Boltzmann/generalized Born surface area (MM-PB/GBSA) continuum solvation models within AMBER. The electrostatic surface of PAMAM dendrimer–nucleic acid complexes were visualized using the DelPhi program.^67^ However, due to the significantly larger size of G7 dendrimers, full data analysis was performed only for G3 to G6 dendrimers.

### Preparation of NANPs

All sequences are listed in the Supporting Information. The DNA strands for DNA cubes and the DNA templates used to transcribe RNA cubes were purchased from Integrated DNA Technologies (Coralville, IA). RNA cube templates were PCR-amplified using MyTaq Mix from Bioline (London, UK), and the resulting PCR products were purified using the DNA Clean and Concentrator kit from Zymo Research (Irvine, CA). T7 RNA polymerase was used for *in vitro* runoff transcription under the following conditions: 80 mM HEPES-KOH (pH 7.5), 2.5 mM spermidine, 50 mM DTT, 25 mM MgCl_2_, and 5 mM rNTPs. The transcription mix was incubated at 37 °C for 3.5 h, followed by the addition of RQ1 RNase-free DNase (Promega, Madison, WI). The RNA products were purified using 15% denaturing polyacrylamide gel electrophoresis (PAGE) containing 8 M urea. RNA bands were visualized under short-wavelength UV light, excised, and eluted in a crush and soak buffer (300 mM NaCl, 89 mM tris-borate (pH 8.2), 2 mM EDTA) overnight. RNA was then precipitated in 2× volumes of 100% ethanol for 3 h at −20 °C. After centrifugation at 14,000 RCF for 30 min, the samples were washed twice with 90% ethanol for 10 min each. The supernatant was discarded, and the samples were vacuum-dried and dissolved in ET-free water. NANPs were synthesized in a one-pot assembly, where purified monomers were combined at equimolar concentrations in double-deionized, ET-free water. The solutions were heated to 95 °C, snap cooled to 45 °C, and assembly buffer (89 mM tris-borate (pH 8.2), 2 mM MgCl_2_, 50 mM KCl) was added after 2 min. The mixtures were incubated at 45 °C for an additional 20 min and then stored at 4 °C for all experiments. DNA duplexes were assembled by combining equimolar amounts of complementary strands (one labeled with Al488) in ET-free water. The samples were heated to 95 °C for 2 min, followed by the addition of 5× assembly buffer at 20% of the final volume. The samples were then incubated at room temperature for 20 min before storage at 4 °C.

### Physicochemical Characterization of NANPs

To analyze the NANPs, 8% nondenaturing native-PAGE (37.5:1) was performed using a buffer containing 89 mM tris-borate (pH 8.2) and 2 mM MgCl_2_. The gels were run at 300 V for 20 min at 4 °C using the Mini-PROTEAN Tetra system (Bio-Rad, Hercules, CA). Then, the gels were washed with double-deionized water and stained with ethidium bromide for 5 min to visualize the assemblies using a ChemiDoc MP system (Bio-Rad, Hercules, CA). Atomic force microscopy (AFM) of DNA and RNA cubes (Figure 2) was conducted on freshly cleaved 1-(3-aminopropyl)­silatrane-modified mica surfaces using a MultiMode AFM Nanoscope IV system (Bruker Instruments, Santa Barbara, CA) in tapping mode.^57^

**Figure 2:**
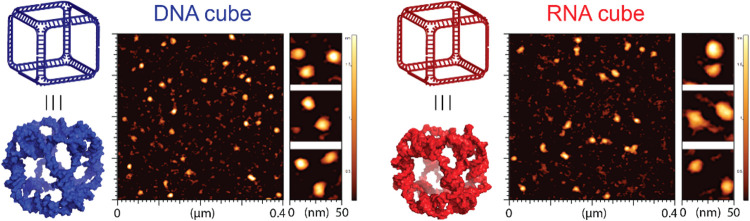
AFM images of RNA and DNA cubes used in this work.

### PAMAM Dendrimers

Amine-terminated PAMAM dendrimers were purchased from Dendritech, Inc. Per the guidelines of the manufacturer, the dendrimer stocks were dried overnight using a SpeedVac system set at 30 °C, followed by lyophilization for approximately 3 h to ensure complete removal of the residual methanol storage solution. The resulting dry pellet was weighed, and the dendrimers were rehydrated to a stock concentration of 1 mg/mL using endotoxin (ET)-free water. For subsequent experiments, the stock solution was diluted in ET-free water to a working concentration of 0.1 mg/mL to maintain consistency across all assays. The 1 mg/mL stock solution was stored at −20 °C until it was needed for experiments. Before use, it was thawed and diluted to 0.1 mg/mL, then stored at 4 °C for up to 1 week before being discarded.

### Binding Assays

The amine-to-phosphate (N/P) ratios were calculated based on the known number of amine groups in each generation of PAMAM dendrimers (which varies with the dendrimer generation, Figure 3C chart) and the number of phosphate groups in the backbone of the respective nucleic acid cargo. For example: *DNA duplex* contains 52 phosphate groups; *DNA cube* contains 306 phosphate groups; and *RNA cube* contains 324 phosphate groups.

**Figure 3:**
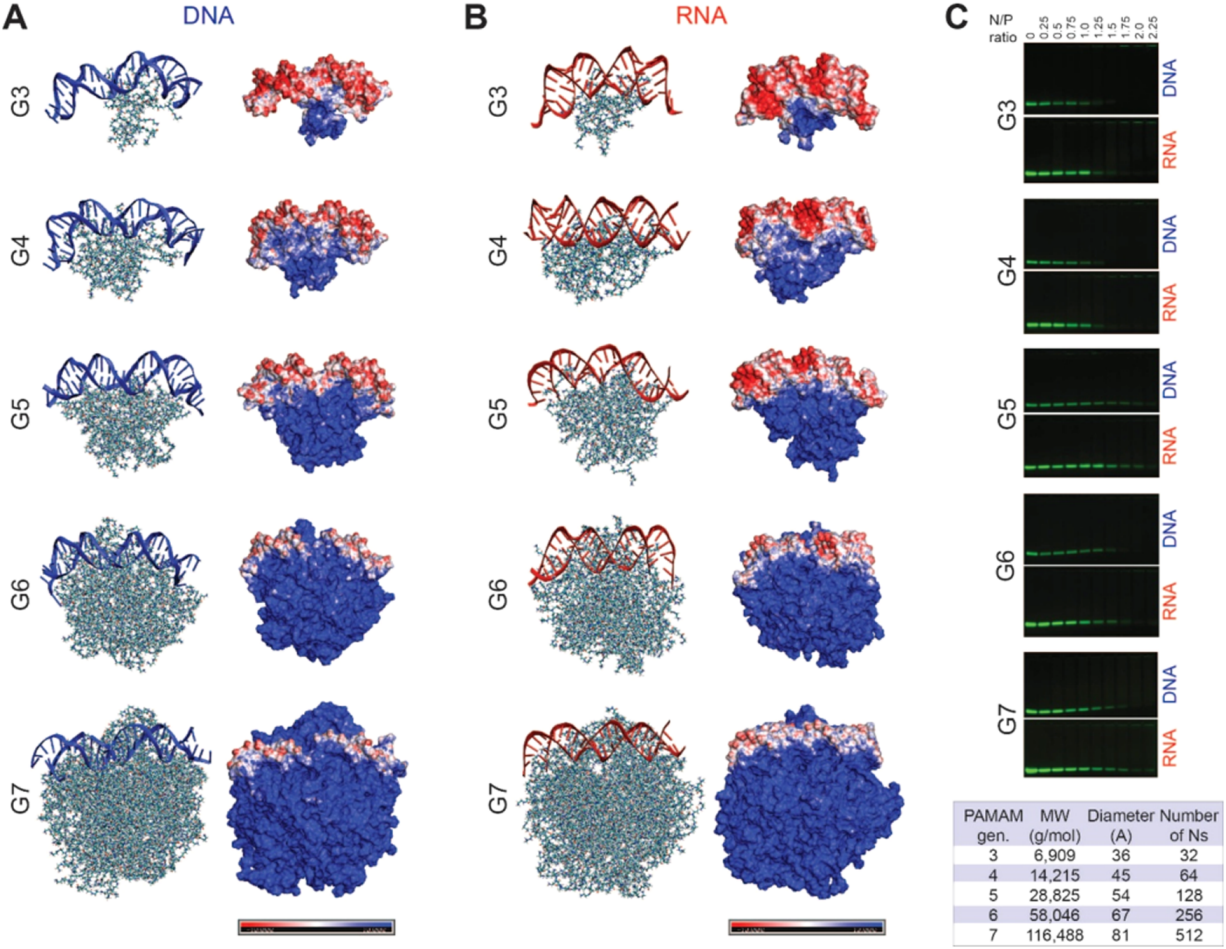
Computational and experimental assessment of nucleic acid duplexes binding with each dendrimer generation. Complex formations of (A) DNA and (B) RNA with dendrimers. Each structure is the MD snapshot at 100 ns. The DNA and the RNA are represented by blue and red helices, respectively. Red, blue, and white regions on the electrostatic potential surfaces represent negatively charged, positively charged, and neutral areas, respectively. (C) Electromobility shift assays of DNA and RNA duplexes binding with each dendrimer generation and description of each dendrimer generation to include their molecular weight, diameter, and number of available amines that can bind with duplexes. Parameters for the summary table are used from Dendritech, Inc.

To evaluate the binding capacity of positively charged PAMAM dendrimers to negatively charged Alexa 488-labeled DNA (or RNA) duplexes, the duplexes were complexed with each dendrimer generation at varying N/P ratios. Before complexation, dendrimers at a concentration of 0.1 mg/mL were vortexed for ∼ 30 s to ensure uniform dispersion. The respective dendrimer and DNA duplex were then combined in an Eppendorf tube, briefly centrifuged, and incubated for 30 min at RT. Following incubation, samples were mixed 1:1 with agarose loading buffer (30% glycerol (v/v) with bromophenol blue and xylene cyanol dyes) to ensure proper loading and migration. The mixtures were then loaded onto a 1.5% agarose gel, electrophoresed for 15–20 min at 200 V and imaged using the ChemiDoc MP system (Bio-Rad).

### Cell Culture Experiments

This study employed well-characterized cell lines available from commercial sources as detailed below.

### Monolayer Cell Uptake and Cell Viability Assessment

Human embryonic kidney cells (HEK-293FT, ATCC CRL-1573) were cultured in a 75 cm^2^ flask using Dulbecco’s Modified Eagle Medium (DMEM) supplemented with 10% heat-inactivated fetal bovine serum (FBS) and 1% penicillin-streptomycin (p/s). Cells were maintained at 37 °C with 5% CO_2_. To detach the cells, 1 mL of 0.25% Trypsin-EDTA was added, and the flask was incubated at 37 °C with 5% CO_2_ for 5 min until complete detachment. The trypsin was then neutralized with 9 mL of complete DMEM, and cell counting was performed. For uptake experiments, cells were seeded at a density of ∼ 50,000 cells per well in a 24-well Greiner plate with a final volume of 480 μL and incubated for 48 h. After the incubation period, cells were transfected with Alexa 488-labeled NANPs and duplexes previously complexed with the respective dendrimer generation. The dendrimers were vortexed before complexation. Then, the NANPs were mixed with the PAMAM dendrimers with an N/P ratio of 2, briefly centrifuged, and then incubated at RT for 30 min. Twenty-four h post-transfection, cellular uptake was visualized using a Leica fluorescence microscope. Following imaging, the supernatant was collected, and 250 μL of 0.25% Trypsin-EDTA was added to each well. Cells were incubated for 5 min at 37 °C with 5% CO_2_ until detachment, after which trypsin was neutralized with an equal volume of complete DMEM. The resulting 500 μL cell suspension was transferred to a 1.5 mL Eppendorf tube. Cells were centrifuged at 700*g* for 5 min at 4 °C, and the supernatant was removed and replaced with 1× PBS supplemented with 4% bovine serum albumin (BSA), 0.2 mM EDTA, and 5 nM Sytox Red cell stain. After a 15 min incubation at room temperature, samples were analyzed using an Attune NxT flow cytometer. The OVERTON analysis tool was employed to determine the percentage of cellular uptake and cell death. L2K (Thermo Fisher Scientific) was used as a control reagent for all experiments with cells. The NANPs and duplexes were complexed with L2K following the manufacturer’s protocol and incubated at RT for 30 min before transfections.

### Spheroids Formation, Uptake, and Cell Viability Assessment

Spheroids were generated using the hanging-drop method.^68^ Commertial monolayer-cultured HEK-293FT (ATCC CRL-1573) and PANC-1 (ATCC CRL-1469) cells were washed with PBS, detached using 0.25% trypsin-EDTA, and assessed for viability and cell count via trypan blue staining. A total of ∼ 2,000 cells were seeded onto the inverted lids of 96-well plates in 20 μL drops of complete medium (DMEM supplemented with 10% heat-inactivated FBS and 1% penicillin-streptomycin). To maintain humidity and prevent evaporation, 100 μL of 1X PBS was added to the well bottoms. The plates were incubated at 37 °C and 5% CO_2_ for 4 days to allow spheroid formation. On day 4, spheroids were carefully collected and transferred to agarose-coated 96-well plates (1% agarose, 60 μL per well) containing 180 μL of complete medium. The following day, spheroids were transfected by adding 20 μL of different generations of dendrimers previously complexed with Al488-labeled DNA duplexes, DNA cubes, or RNA cubes. The dendrimers were vortexed before complexation. Then, the NANPs were mixed with the PAMAM dendrimers with an N/P ratio of 2, briefly centrifuged, and then incubated at RT for 30 min. On day 6, 24 h post-transfection, spheroids were imaged using a Leica fluorescence microscope before and after washing. For flow cytometry, each sample graphed is comprised of nine individually grown and transfected spheroids, wherein three spheroids were combined to create one technical repeat, allowing for a gated 10,000 event. A biological repeat equates to three repeats of the previously described process. After combining, the spheroids were dissociated with 0.25% Trypsin-EDTA and stained with 5 nM Sytox-Red in 1X PBS (supplemented with 0.2 mM EDTA and 4% BSA) for 15 min at room temperature to assess cell death using an Attune NxT flow cytometer. Dendrimer uptake (measured by the percentage of Al488-positive cells) and cell death were analyzed using Attune software with the Overton analysis tool.

### OrganoPlate Culture, TEER Analysis, and Imaging

OrganoReady Colon Caco-2 (Mimetas BV, The Netherlands) plates were used for the assessment of the uptake of fluorescently labeled NANPs in 3D culture. The plate includes 64 Caco-2 tubules seeded against collagen I as previously described.^69,70^ The Caco-2 tubules are leak-tight and can be used to assess the barrier integrity of the 3D. Al488-labeled NANPs were complexed with five generations of PAMAM dendrimers (G3-G7) and transfected into Caco-2 tubules. The dendrimers were vortexed before complexation. Then, the NANPs were mixed with the PAMAM dendrimers with an N/P ratio of 2, briefly centrifuged, and then incubated at RT for 30 min before completing to the final volume with media. The final concentrations of NANPs tested were 20 and 50 nM. All samples were tested in triplicates. Before transfection, all inlets and outlets of the chip were aspirated, then 50 μL of treatment was added to each right inlet and outlet. The OrganoPlate was placed on the OrganoFlow rocker (Mimetas BV, The Netherlands) at 14°/8 min settings in a 37 °C, 5% CO_2_ incubator for 24 h.

The transepithelial electrical resistance (TEER) values measure the barrier integrity of the Caco-2 tubules. This method provides accurate and sensitive measurements of the tightness of the monolayer of the tubules. It can be used to assess the toxicity of the treatments to the cells, where their induced disruption can be measured at the end point of the experiment. In this study, the transfected NANPs were tested for their effect on the barrier integrity. Staurosporine (33 nM), which is a protein kinase inhibitor, was used as a positive control, as it disrupts the barrier integrity significantly. All OrganoPlates were equilibrated at room temperature for 30 min before TEER measurements. Before transfection (t = 0), the TEER of the chips was measured and recorded using the OrganoTEER (Ω*cm^2^) (Mimetas BV, The Netherlands). Twenty-four hr after treatment, the TEER was measured again and recorded for analysis. At the end point of the experiment, 24 h after transfection, the cells were washed and fixed with 3.7% formaldehyde (Sigma) in HBSS with calcium and magnesium (Gibco) for 15 min, washed twice with PBS (Gibco) for 5 min and then stored with 50 μL PBS per well at 4 °C until imaging. The OrganoPlate was imaged using a Cytation 5 Multi-Mode Microplate Reader (BioTek) using the GFP filter at 4×. Images were processed with Fiji.

### Immunostimulation Assessed in Reporter Cell Lines

Engineered human embryonic kidney cell line HEK-Lucia RIG-I and human acute monocytic leukemia cell line THP1-Dual were obtained from InvivoGen and maintained at 37 °C with 5% CO_2_ in accordance with InvivoGen’s protocols. Cells were seeded in a 96-well flat-bottom Greiner plate, with HEK-Lucia RIG-I cells plated at ∼50,000 cells per well and THP1-Dual cells at ∼ 100,000 cells per well. Transfections were performed using dendrimer carriers complexed with NANPs, duplexes, or positive controls. Positive controls included RNA cube (10 nM), PAM3CSK (6 μg/mL), and 2′3′-cGAMP (1 μg/mL). RNA cube and 2′3′-cGAMP were preincubated with L2K for 30 min before transfection. Post-transfection, cells were incubated at 37 °C with 5% CO_2_ for 24 h prior to evaluating SEAP activation, IRF activation, and viability. For HEK-Lucia RIG-I cells, IRF activation was assessed using a QUANTI-Luc assay. THP1-Dual cells were evaluated using both a QUANTI-Blue assay to measure SEAP activation and a QUANTI-Luc assay for IRF activation. Cell viability was determined post-transfection using an MTS colorimetric assay following the manufacturer’s guidelines. Absorbance measurements were taken using a Tecan Spark plate reader. All samples were normalized to untreated cells, and data were collected from three biological replicates (N = 3) performed in triplicate.

## Results and Discussion

### MD Simulations

MD simulations visualized nucleic acid interactions with PAMAM dendrimers (Figure 3). At 100 ns, DNA-bound G3 dendrimers partially, leaving eight base pairs unbound, whereas RNA adhered more fully due to its flexibility. G4 dendrimers facilitated uniform binding, with protonated amines interacting differently with DNA (minor/major grooves) and RNA (backbone). G5 and G6 dendrimers progressively increased nucleic acid surface coverage, with G6 nearly saturating both RNA and DNA surfaces (SI Figure S1). Surface coverage analysis (SI Figure S2A) showed a steady increase from G3 to G5, peaking with G6. DNA exhibited minimal coverage with G3, moderate increases in coverage with G4 and G5, and a sharp rise with G6. Electrostatic potential analysis indicated that at low N/P ratios, nucleic acids promoted additional dendrimer binding, while at high N/P ratios (*e*.*g*., for G6), positively charged amines dominated, enabling multiple nucleic acid attachments. Hydrogen bonding (HB) analysis (SI Figure S2B) revealed distinct binding patterns. RNA-dendrimer HB steadily increased with size, whereas DNA exhibited a higher number of HB interactions for G4 when compared to G5. G6 complexes showed the highest HB interactions. Electrostatic interactions (SI Figure S3) confirmed that G6 dendrimers induced denser amine-DNA interactions due to B-form DNA’s narrower minor groove. Binding free energy calculations (SI Table S2) showed G3 dendrimers bound RNA duplexes stronger than DNA counterparts. For G4 and G5 dendrimers, MM-PBSA and MM-GBSA produced inconsistent trends, likely due to intermediate dendrimer sizes. G6 simulations consistently indicated stronger binding to DNA duplexes, attributed to structural differences favoring electrostatic interactions. G7 dendrimers exhibited the strongest electrostatic interactions with duplexes (Figure 3). Interestingly, despite these strong interactions, nucleic acids associated with G7 dendrimers underwent significantly less deformation, likely due to the dendrimers’ lowest curvature, when compared to G3-G6 analogs. The root-mean-square deviation (RMSD) values for DNA (4.8 Å) and RNA (3.3 Å) duplexes complexed with G7 dendrimers were lower than those observed with G6 dendrimers-DNA (6.6 Å) and -RNA (5.2 Å) complexes. These results suggest that G7 dendrimers can deliver nucleic acid cargos with reduced structural deformation compared to lower generation dendrimers.

Overall, MD simulations demonstrated that dendrimer size and N/P ratio critically influenced nucleic acid binding, with larger dendrimers providing greater nucleic acid surface coverage and a higher number of HB.

The unique biophysical properties of dendrimers as nucleic acid delivery agents were further examined through comparisons with alternative carriers such as bola amphiphiles.^15,65,66^ Bolaamphiphiles possess two positively charged head groups connected by a hydrophobic alkyl chain and can aggregate to form micelles or vesicles due to strong hydrophobic interactions. The positive charge of the head groups allows negatively charged nucleic acids to associate with the bolaamphiphile surface. However, when bolaamphiphiles form micelles, the rigid hydrophobic core prevents the rearrangement of positively charged head groups from optimizing interactions with RNA.^64^ Compared to bolaamphiphile carriers, dendrimers’ structural flexibility enhanced nucleic acid interactions, suggesting superior binding affinity and protection for nucleic acid delivery.

### Binding Assays

Dendrimer-nucleic acid interactions were assessed through electromobility shift assays (EMSA) using Alexa 488-labeled DNA and RNA duplexes (Figure 3C and SI Figure S4). Complex formation was examined by maintaining a constant concentration of fluorescently tagged duplexes while incrementally increasing dendrimer amounts to achieve different N/P ratios. Samples were then analyzed using 1.5% (w/v) agarose gel electrophoresis. Uncomplexed duplexes migrated freely through the gel, whereas those electrostatically bound to dendrimers exhibited reduced mobility, with some becoming entirely retained in the wells. The N/P ratio required for complete complexation varied by dendrimer generation. Specifically, full retardation was observed at N/P ratios of ∼1.25–1.5 for G3, ∼1.25–1.5 for G4, ∼2 for G5, ∼1.5–1.75 for G6, and ∼1.75–2 for G7 dendrimers with no significant differences in bindings to RNA and DNA duplexes. To ensure complete dendrimer-nucleic acid complexation, an N/P ratio of 2 was selected for downstream experiments for all generations. This ratio was chosen as a conservative threshold to ensure robust nucleic acid complexation across all dendrimer generations.

### Influence of Dendrimer Generation on NANPs Uptake in Traditional 2D Cell Cultures

Using monolayer cell cultures, we assessed uptake after a 24-h transfection with Alexa 488-labeled NANPs and duplexes complexed with each generation of dendrimers. Microscopy images revealed that all dendrimer generations complexed with DNA or RNA cubes were internalized by HEK-293FT cells grown in monolayers (Figure S5A), with G6-complexed RNA cubes exhibiting the highest accumulation. Interestingly, L2K showed the lowest fluorescence for RNA cubes, suggesting that dendrimers were more efficient in delivering this particular NANP type. In contrast, DNA duplexes combined with L2K were internalized by cells, whereas dendrimers did not, regardless of the generation. These findings are in accordance with flow cytometry data (Figure S5B). Notably, while flow cytometry showed similar uptake percentages for both RNA and DNA cubes delivered by dendrimers and L2K, microscopy revealed that although the percentage of cells that internalized NANPs was similar between dendrimers and L2K, the fluorescence intensity per cell was higher with dendrimer delivery. Our findings suggest that dendrimers may enhance overall intracellular NANP uptake in monolayer cells. In terms of cytotoxicity, no significant increase in cell death was observed across treatments, indicating that dendrimer-mediated delivery did not compromise the viability of HEK monolayers.

### Influence of Dendrimer Generation on NANPs Uptake in 3D Spheroids

NANPs uptake by cells is influenced by multiple factors, including carrier features, NANP structure, and the cellular environment. In tridimensional culture models, the spatial organization of receptors and the accessibility of nanoparticles to cells are different than in a monolayer cell models. In spheroids, receptor accessibility is likely altered due to the different cell–cell interactions and extracellular matrix components, which may impact the efficiency and mechanism of NANP internalization, as well as imposing penetration barriers to the spheroids’ core. The 3D spheroid models more accurately mimic the complexity of tissues *in vivo*, and as such, we analyzed the delivery efficiency of NANPs in HEK-293FT, and tumoral PANC-1 spheroids. These cell lines were chosen for their consistent ability to form spheroids with uniform size, density, and cell number. Additionally, we aimed to compare noncancerous and cancerous cell lines to evaluate potential differences in uptake and cell viability for dendrimer-NANP formulations. As shown in microscopy images, 24 h post-transfection (Figure 4A–B), for PANC-1 spheroids, G3 dendrimers exhibited the highest efficiency for duplex delivery, with an uptake of approximately 12%. G3 is the smallest dendrimer and showed better delivery for duplexes than for NANPs. In contrast, G4 and G5 were more effective for NANP delivery, reaching around 15% uptake for both RNA and DNA cubes, with no significant difference between nucleic acid types. For G6 and G7 dendrimers, DNA cubes exhibited the highest uptake. When using L2K, the most efficiently delivered NANP was the DNA cube (∼20%), followed by the DNA duplex (∼15%), while the RNA cube had the lowest uptake (∼8%). In HEK spheroids (Figure 4C–D), G4-G7 dendrimers preferentially delivered RNA cubes, achieving approximately 20% uptake, whereas DNA cubes and duplexes were less efficiently taken up. For L2K, the DNA duplex showed the highest uptake (∼30%), with no significant difference between RNA and DNA cubes. Interestingly, G3 and G4 dendrimers with cubes were the most toxic to HEK spheroids, inducing 20–30% cell death, while in PANC spheroids, cell death varied from ∼8–15%, without a clear correlation with NANP type or dendrimer generation.

**Figure 4:**
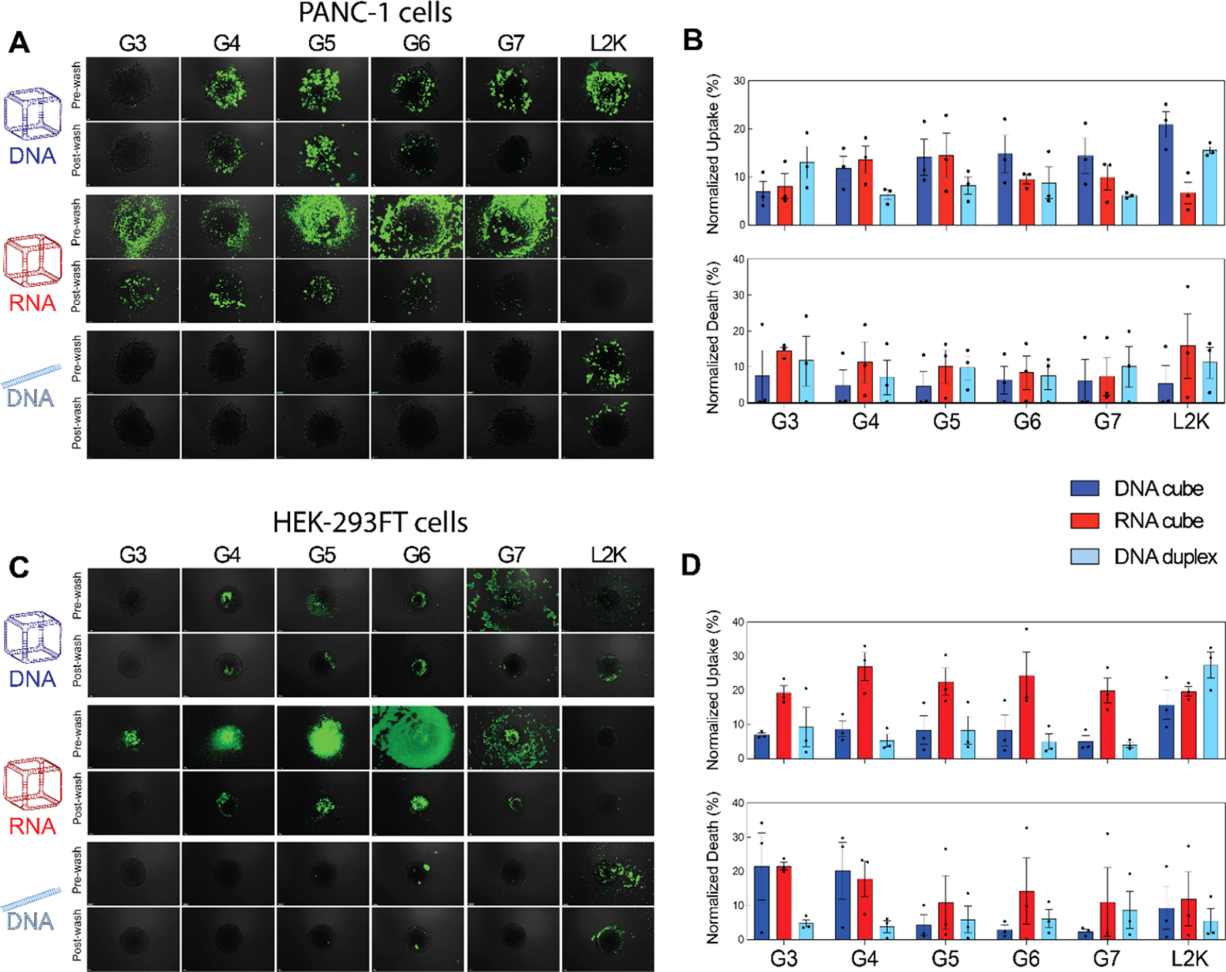
Evaluation of cellular uptake and cell death in PANC-1 and HEK-293FT using 3D model spheroids. (A) Merged microscopy images of PANC-1 spheroids treated with varying NANP-dendrimer complexes. (B) OVERTON flow cytometry analysis of cellular uptake and cell death in PANC-1 spheroids following treatment with NANP-dendrimer complexes. (C) Merged microscopy images of HEK-293FT spheroids treated with NANP-dendrimer complexes. (D) OVERTON flow cytometry analysis of cellular uptake and cell death in HEK-293FT spheroids following treatment with NANP-dendrimer complexes. In (A and C), scale bar = 100 μM. In (B and D), *N* = 3, Mean ± SEM.

Figure 4A,C show representative fluorescence images for PANC and HEK spheroids, respectively. Markedly, observations of the extracellular environment can be made prior to washing the spheroids. For example, dendrimers complexed with NANPs accumulated around and on the surface of the spheroids and exhibited the highest fluorescence intensity across all dendrimer generations. However, after washing the spheroids, only G3-G6-complexed NANPs remained visibly bound to the spheroids in both cell lines. For DNA cubes, complexation with G4-G6 dendrimers consistently showed high fluorescence both pre- and postwash, suggesting stable binding and uptake. Interestingly, DNA duplexes exhibited minimal fluorescence both before and after washing, suggesting a different uptake mechanism. The flow cytometry data (Figure 4B,D, top panels) indicate that duplexes had measurable internalization despite low spheroid surface fluorescence, likely due to their small size, allowing deeper penetration into the spheroid core rather than accumulating at the surface.

### OrganoPlates

New approach methodologies (NAMs), including 3D cell cultures within microphysiological systems, offer additional biological relevance over conventional 2D techniques and *in vivo* models, as highlighted by the recent FDA announcement in favor of more NAM-generated data in investigational new drug applications (fda.gov). Therefore, to further assess the relative uptake in 3D culture, Caco-2 tubules were cultured within an OrganoPlate 3-lane 64. Caco-2 cells were chosen as proof-of-concept because as polar, differentiated epithelial cells, they historically demonstrate low transfection efficiency,^71^ thus necessitating the use of novel delivery platforms and in turn, relevant models with which to evaluate these new drug modalities.^72^ Their cultivation and differentiation is also greatly enhanced with the use of 3D over 2D culture.^73^ Each chip is comprised within a 2 × 3 block of wells in a standard 384-well plate. The chip consists of the gel channel (A2), right channel inlet and outlet (A3, B3), left channel inlet and outlet (A1, B1), and the observation window where all three channels intersect (B2) for imaging. For the Caco-2 model, collagen-I is first seeded in A2, then cells are added to the right channel inlet (B3) and media is added to A1, B1, A3, and B3. As a result, upon perfusion, the cells form 3D tubules in the right channel, as shown in the 3D drawing (Figure 5A). Caco-2 OrganoPlates were transfected with dendrimer-NANP complexes to assess relative uptake and effect on transepithelial electrical resistance (TEER) after 24 h. As seen in Figure 5B, the uptake of NANPs was observed to increase and correlate with increasing generations of PAMAM dendrimers, with G7 having the highest uptake for the Alexa 488-labeled RNA cubes and DNA cubes as compared to the rest of the PAMAM dendrimers generations tested. In line with the HEK-293FT spheroid model, where RNA cubes exhibited the highest uptake, the Caco-2 tubule model also demonstrated preferential uptake of RNA cubes over DNA cubes. Similarly, PANC-1 spheroids showed uptake of both DNA and RNA cubes, aligning with the Caco-2 model, where both were internalized, but RNA cubes displayed superior uptake. However, unlike with spheroid cultures, there was no loss of fluorescent signal after fixation of the OrganoPlate, perhaps as a result of the continuous bidirectional perfusion of the culture during uptake. The TEER measurements (Figure 5C) show that none of the treatments or the vehicle controls disrupted the barrier integrity of the Caco-2 tubules after 24 h of transfection, as compared to the positive control, indicating that neither the NANPs complexed with PAMAM dendrimers or L2K, nor the vehicle controls alone were cytotoxic. Fluorescence imaging also demonstrated that the uptake of NANPs was concentration-dependent, as an increase in uptake was shown for 50 nM NANPs (Figure 5B) as compared to 20 nM NANPs (SI Figure S6). This dependency was confirmed with the images where L2K was used as a carrier. Quantitative analysis of fluorescent intensity, as an indicator of NANPs’ uptake, demonstrated comparable signal between G3- and G4- complexed particles. While the fluorescent signal was stronger when NANPs were complexed with the higher generation dendrimers, there was no difference between G5-, G6- and G7- complexed samples.

**Figure 5:**
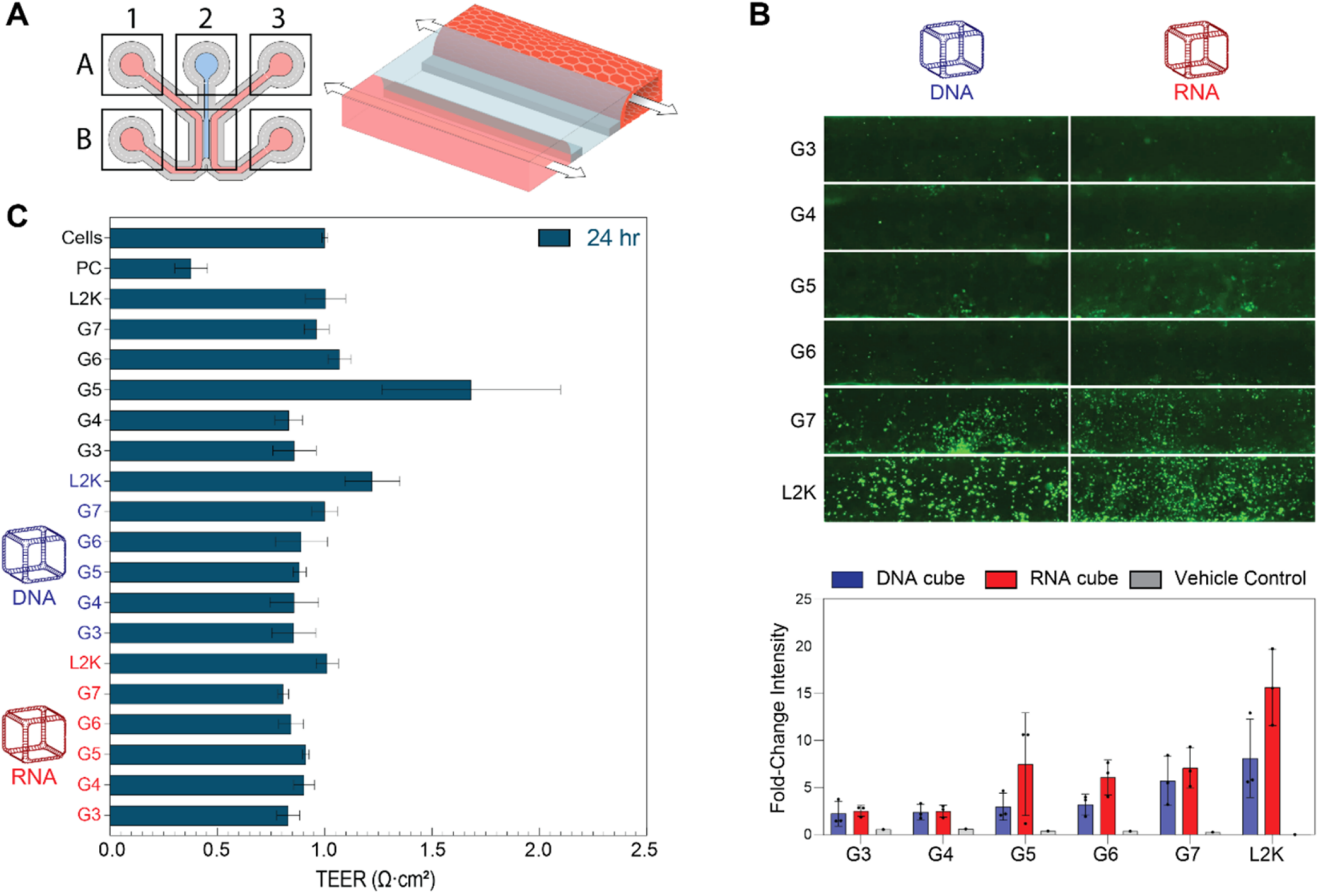
TEER analysis and microscopy images of Caco-2 cultures transfected with dendrimer-nucleic acid complexes. (A) Layout of a 3L64 OrganoReady Colon Caco-2 chip, and 3D drawing showing the Caco-2 tubule seeded against the collagen-I. (B) Representative microscopy images of OrganoPlate cultures at 4× magnification showing fluorescent uptake of dendrimer-NANP complexes for 24 h. The microscopy images show the uptake of NANPs within the Caco-2 3D tubule, where the uptake increases as the generation of dendrimers increases from G3-G7. The intensity of the Al488 signal was quantified and normalized to the cells-only control, presented as mean ± SD, (*n* = 3) (**C**) Transepithelial electrical resistance (TEER) measurements following treatment with DNA or RNA cubes complexed with different generations of dendrimers. Control conditions include vehicles alone and positive control that disrupts the barrier integrity. TEER values are normalized against *t* = 0, and presented as mean ± SEM, (*n* = 3).

### Immune Reporter Cell Lines

In HEK-Lucia RIG-I cells, L2K complexed with RNA cubes served as a positive control, as established in previous studies.^17^ As shown in Figure 6A, L2K-complexed RNA cubes induce significant IRF activation. However, when RNA cubes are complexed with varying generations of PAMAM dendrimers (G3–G7), no significant IRF activation is observed, which is consistent with the results of our earlier study demonstrating that unlike L2K, complexation of NANPs with G5 amine-terminated dendrimers eliminated secreted type I and type III interferons in PBMCs.^8^ The observed lack of significant IRF activation in the presence of dendrimer-RNA cube complexes suggests that PAMAM dendrimers may not be as effective as L2K in facilitating NANPs uptake into and the dynamics of the complex behavior in the cellular compartment promoting RIG-I recognition of RNA in HEK-Lucia RIG-I cells. This may be due to the lower delivery efficiencies when compared to L2K and strong electrostatic binding between the cationic dendrimers and RNA cubes, which could potentially continue shielding the RNA cubes from interaction with the cytosolic innate immune sensor RIG-I. However, the slight increase in activation (more than 2-fold above the baseline for G6) suggests that higher-generation PAMAM dendrimers may promote the uptake and cytosolic appearance of the RNA cubes, enabling some degree of recognition by RIG-I; however, additional mechanistic studies would be required to verify it. The slight activation observed in higher-generation dendrimers may also suggest a threshold effect, where dendrimer properties (*e*.*g*., size, surface charge) need to reach a critical point to effectively deliver NANPs at levels sufficient to trigger immune activation. Furthermore, the higher density of terminal surface groups, suggested by MD simulations, and imperfect surface functionality in higher dendrimer generations may limit their ability to complex with NANPs, contrary to expectations based on the doubling of molecular weight and surface group numbers with each generation. In reality, higher-generation dendrimers often deviate from the expected number of surface groups due to increased branching density, a limitation described by the De Gennes dense packing phenomenon, and exhibit reduced complexation efficiency as a result of steric hindrance.^74^ As expected, IRF activation was not observed in RIG-I cells when treated with DNA particles, since RIG-I is not a pattern recognition receptor responsive to DNA. Thus, no IRF activation is seen with DNA cubes or DNA duplexes.

**Figure 6:**
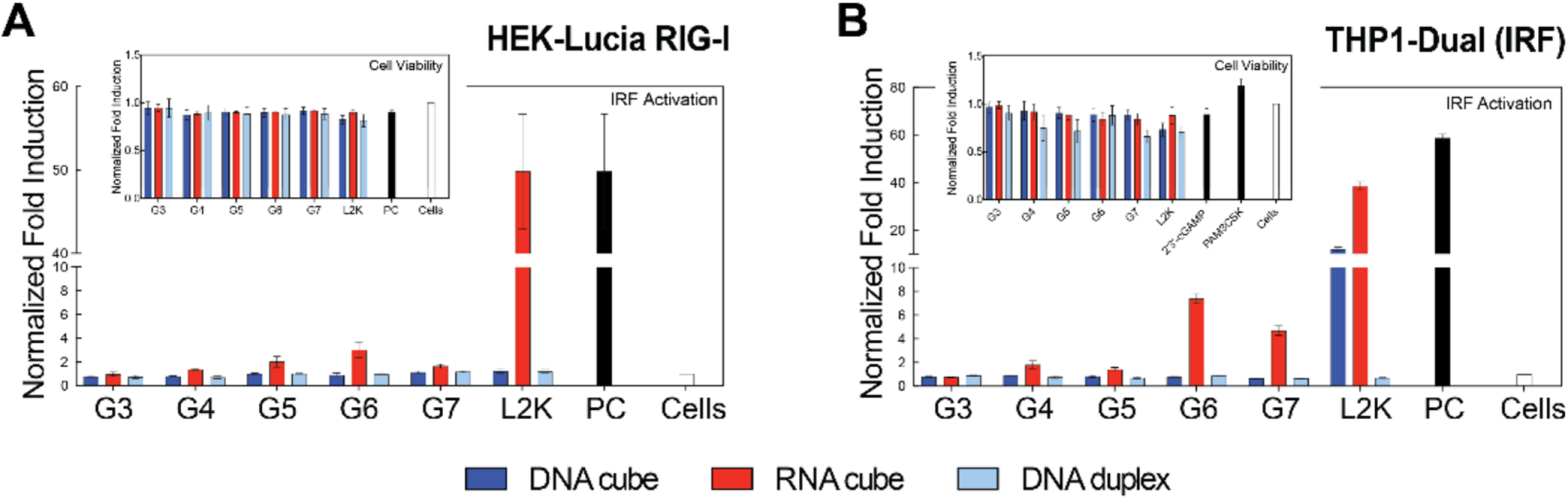
Assessment of immune activation and cell viability to DNA cubes, RNA cubes, and DNA duplexes complexed with dendrimers. Normalized fold induction of IRF activation using (A) HEK-Lucia RIG-I and (B) THP1-Dual cells. Note that PC in (A) is the same as L2K/RNA cube sample. Within each graph, there is a subset of the normalized cell viability for each cell line after transfected with each sample. (*N* = 3, Mean ± SEM). THP-1 Dual cells were also tested for NF-κB activation in each sample (data shown in the SI).

Furthermore, when dendrimers are used to deliver NANPs into THP1-Dual cells, as shown in Figure 6B, IRF activation is observed with L2K-transfected RNA cubes (also seen in RIG-I cells) and some for DNA cubes, as expected. Interestingly, RNA cubes complexed with G6 and G7 dendrimers also induce activation, highlighting the potential of higher-generation dendrimers in this context. Importantly, no evidence suggests activation via endosomal receptors, as indicated by the lack of SEAP activation across all treatments, confirming that the observed IRF activation is not due to engagement of the endosomal pathway. The activation of IRF in THP1-Dual cells when treated with L2K-complexed RNA cubes aligns with previous findings in RIG-I cells, where L2K is a well-established transfection agent that facilitates efficient RNA delivery and subsequent RIG-I activation. Additionally, some activation is observed in L2K-complexed DNA cube treatments, suggesting involvement of the cGAS-STING signaling pathway.^17^ Interestingly, there is a heightened response seen with RNA cubes complexed with G6 and G7 dendrimers, indicating that higher-generation dendrimers may also contribute to immune activation, potentially via improved cellular uptake and RNA release. The lack of significant IRF activation with lower-generation dendrimers (G3–G5) may imply that these dendrimers are less effective in promoting the necessary cytosolic delivery or RNA exposure to trigger RIG-I-mediated pathways.

The absence of SEAP activation across all treatments (Figure S7) strongly suggests that the observed IRF activation is RIG-I-mediated and not due to the engagement of endosomal pattern recognition receptors. This suggests that the dendrimer-complexed RNA is likely escaping the endosome and reaching the cytosol, where RIG-I can detect it. The observed trend with higher-generation dendrimers underscores the need for further investigation into dendrimer-mediated RNA release, particularly examining the relationship between dendrimer size, surface charge, and nucleic acid cargo.

## Conclusions

Our investigation into amine-terminated PAMAM dendrimers (G3–G7) complexed with DNA and RNA NANPs provided key insights into their structure–activity relationships and immune responses. We found that the dendrimer generation influenced NANP characterization, with variations in how different NANPs interacted with the same dendrimer generation and vice versa. This highlights the intricate interplay between dendrimer properties and NANP behavior, which is critical for their efficacy and immune recognition. In contrast to earlier reports on uncomplexed dendrimers or dendrimers complexed with traditional nucleic acids (plasmid DNA or siRNA), our study showed no significant cytotoxicity in HEK-293FT cells, even with higher-generation dendrimers (e.g., G6 and G7). This suggests that the electrostatic complexation of dendrimers and NANPs effectively neutralizes cationic groups on the dendrimer surface, thereby mitigating dendrimer-associated cytotoxicity. Further studies are needed to explore the mechanisms underlying this effect and their therapeutic implications. Immune activation analysis revealed that RNA cubes complexed with G6 and G7 dendrimers enhanced IRF activation in THP1-Dual cells, likely due to the improved RNA uptake by the cells. These findings provide a deeper understanding of dendrimer-NANP interactions and their implications for nanoparticle-based therapeutics.

## Supplementary Material



## Notes

A list of all nucleic acid sequences used in this work, MD simulation, 2D cell culture experiments, and reporter cell line assays (PDF)
