# Supplementary material for: Changes in Generations of PAMAM Dendrimers and Compositions of Nucleic Acid Nanoparticles Govern Delivery and Immune Recognition
Source: ACS Biomater Sci Eng. 2025 May 20;11(6):3726–37. doi: 10.1021/acsbiomaterials.5c00336 (PMC12152843; doi:10.1021/acsbiomaterials.5c00336)
Supplement: Supplementary file 1 [file ab5c00336_si_001.pdf]

SUPPORTING INFORMATION

Changes in Generations of PAMAM Dendrimers and Compositions of Nucleic Acid Nanoparticles Govern Delivery and Immune Recognition

Yelixza I. Avila[#1], Laura P. Rebolledo[#1], Nathalia Leal Santos[1,2], Brandon Rawlins[1], Yasmine Radwan[1,3], Melanie Andrade-Munoz[1], Elizabeth Skelly[1], Morgan R. Chandler[3], Luciana N. S. Andrade[2], Tae Jin Kim[4], Marina A. Dobrovolskaia[5], Kirill A. Afonin[1]*.

1 – Department of Chemistry, University of North Carolina at Charlotte, Charlotte, NC 28223, USA.
2 - Center for Translational Research in Oncology (LIM24), Instituto do Câncer do Estado de São Paulo, Hospital das Clínicas da Faculdade de Medicina da Universidade de São Paulo, Comprehensive Center for Precision Oncology, Universidade de São Paulo, São Paulo 01246-000, Brazil.
3 – MIMETAS US, INC, Gaithersburg, Maryland 20878, USA.
4 – Department of Physical Sciences, West Virginia University Institute of Technology, Beckley, West Virginia 25801, USA.
5 - Nanotechnology Characterization Laboratory, Cancer Research Technology Program, Frederick National Laboratory for Cancer Research, Frederick, MD 21702, USA

#-these authors contributed equally to this project

*- correspondence to Kirill A. Afonin at kafonin@charlotte.edu

SEQUENCES USED IN THIS WORK

One strand per NANP was modified with an Alexa 488 label to assess uptake by fluorescence.

Six-stranded DNA cube:

5'-GGCAACTTTGATCCCTCGGTTTAGCGCCGGCCTTTTCTCCCACACTTTCACG
5'-GGGAAATTTCGTGGTAGGTTTTGTTGCCCGTGTTTCTACGATTACTTTGGTC
5'-GGACATTTTCGAGACAGCATTTTTTCCCGACCTTTGCGGATTGTATTTTAGG
5'-GGCGCTTTTGACCTTCTGCTTTATGTCCCCTATTTCTTAATGACTTTTGGCC
5'-GGGAGATTTAGTCATTAAGTTTTACAATCCGCTTTGTAATCGTAGTTTGTGT
5'-GGGATCTTTACCTACCACGTTTTGCTGTCTCGTTTGCAGAAGGTCTTTCCGA

Six-stranded fluorescently labeled DNA cube:

5'-GGCAACTTTGATCCCTCGGTTTAGCGCCGGCCTTTTCTCCCACACTTTCACG
5'-GGGAAATTTCGTGGTAGGTTTTGTTGCCCGTGTTTCTACGATTACTTTGGTC
5'-GGACATTTTCGAGACAGCATTTTTTCCCGACCTTTGCGGATTGTATTTTAGG
5'-GGCGCTTTTGACCTTCTGCTTTATGTCCCCTATTTCTTAATGACTTTTGGCC-Alexa 488
5'-GGGAGATTTAGTCATTAAGTTTTACAATCCGCTTTGTAATCGTAGTTTGTGT
5'-GGGATCTTTACCTACCACGTTTTGCTGTCTCGTTTGCAGAAGGTCTTTCCGA

Six-stranded RNA cube:

5'-GGCAACUUUGAUCCCUCGGUUUAGCGCCGGCCUUUUCUCCCACACUUUCACG
5'-GGGAAAUUUCGUGGUAGGUUUUGUUGCCCGUGUUUCUACGAUUACUUUGGUC
5'-GGACAUUUUCGAGACAGCAUUUUUUCCCGACCUUUGCGGAUUGUAUUUUAGG
5'-GGCGCUUUUGACCUUCUGCUUUAUGUCCCCUAUUUCUUAAUGACUUUUGGCC
5'-GGGAGAUUUAGUCAUUAAGUUUUACAAUCCGCUUUGUAAUCGUAGUUUGUGU
5'-GGGAUCUUUACCUACCACGUUUUGCUGUCUCGUUUGCAGAAGGUCUUUCCGA

Six-stranded fluorescently labeled RNA cube:

5'-GGCAACUUUGAUCCCUCGGUUUAGCGCCGGCCUUUUCUCCCACACUUUCACG
5'-GGGAAAUUUCGUGGUAGGUUUUGUUGCCCGUGUUUCUACGAUUACUUUGGUC
5'-GGACAUUUUCGAGACAGCAUUUUUUCCCGACCUUUGCGGAUUGUAUUUUAGG
5'-GGCGCTTTTGACCTTCTGCTTTATGTCCCCTATTTCTTAATGACTTTTGGCC-Alexa 488
5'-GGGAGAUUUAGUCAUUAAGUUUUACAAUCCGCUUUGUAAUCGUAGUUUGUGU
5'-GGGAUCUUUACCUACCACGUUUUGCUGUCUCGUUUGCAGAAGGUCUUUCCGA

Double-stranded DNA duplex:

5'- CGGTGGTGCAGATGAACTTCAGGGTCA
5'- ACCCTGAAGTTCATCTGCACCACCG

Double-stranded fluorescently labeled DNA duplex:

5'- TGACCCTGAAGTTCATCTGCACCACCG-Alexa488
5'- CGGTGGTGCAGATGAACTTCAGGGTCA

SUPPORTING FIGURES

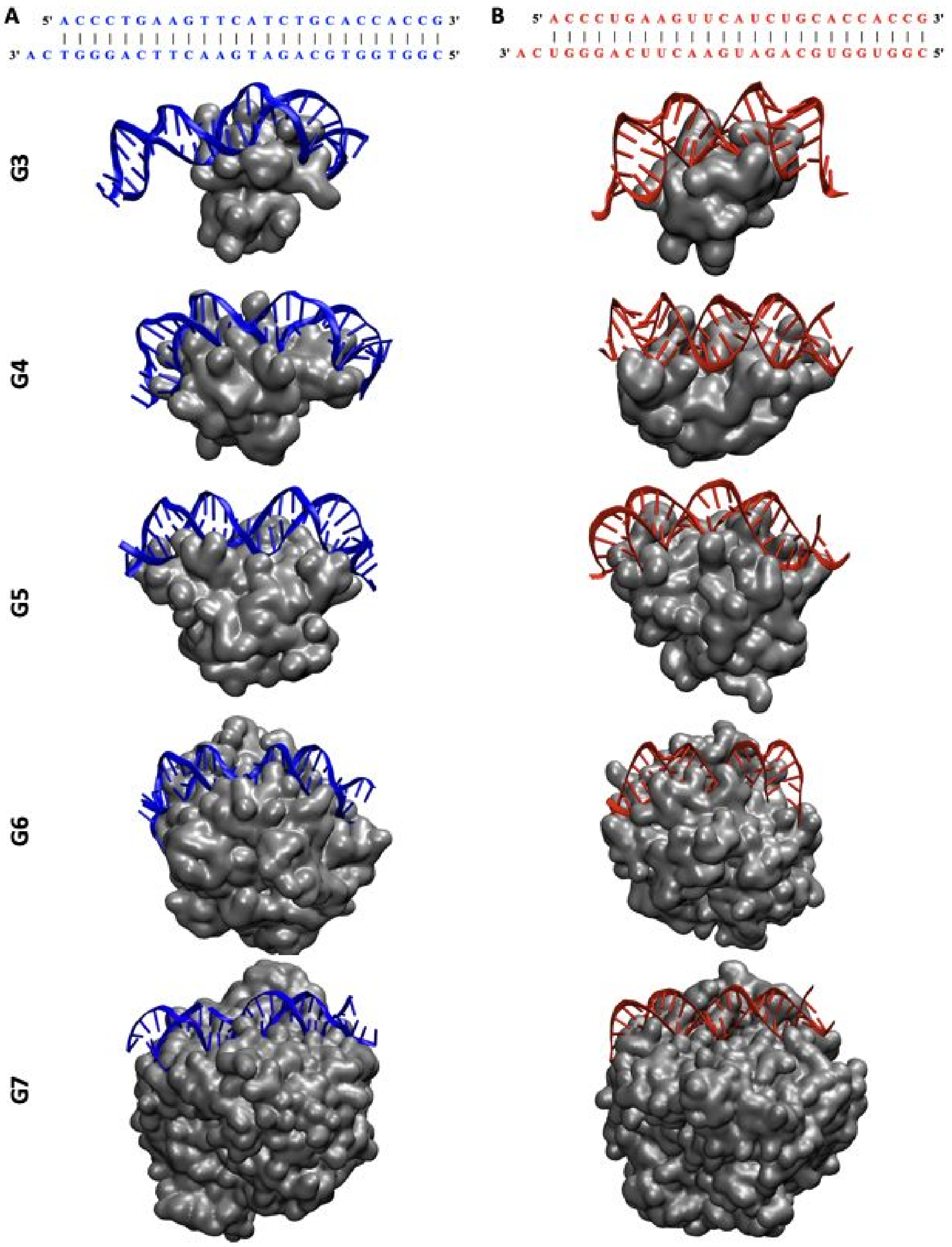

Figure S1. Sequences of (**A**) DNA (blue) and (**B**) RNA (red) used in the MD simulations. Complex formations with each respective PAMAM dendrimer generation (G3–G7) are shown. Dendrimers are visualized using the QuickSurf rendering method in Visual Molecular Dynamics (VMD). DNA and RNA are depicted as blue and red helices, respectively.

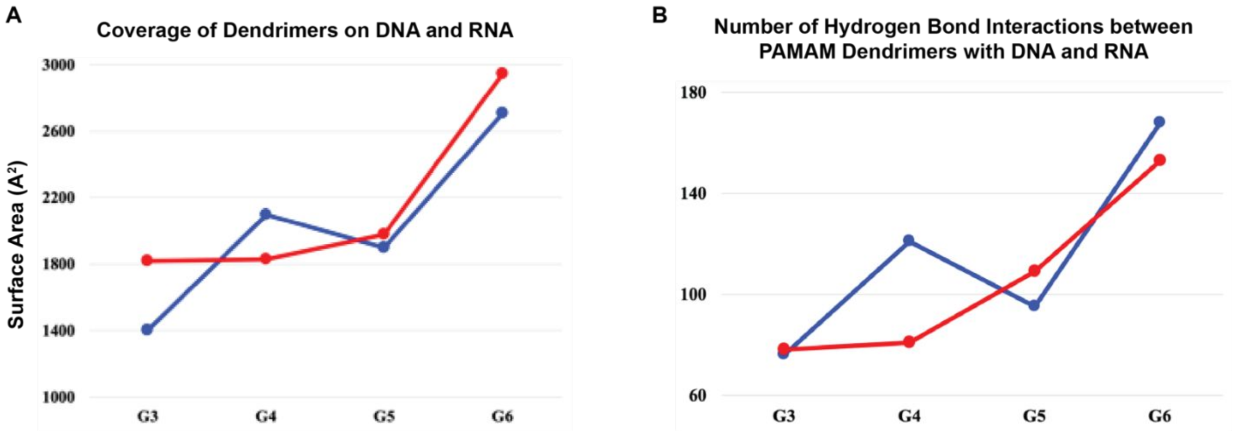

Figure S2. (**A**) Surface area of DNA (blue) and RNA (red) covered by PAMAM dendrimers. (**B**) Number of hydrogen bond interactions formed between PAMAM dendrimers and nucleic acids.

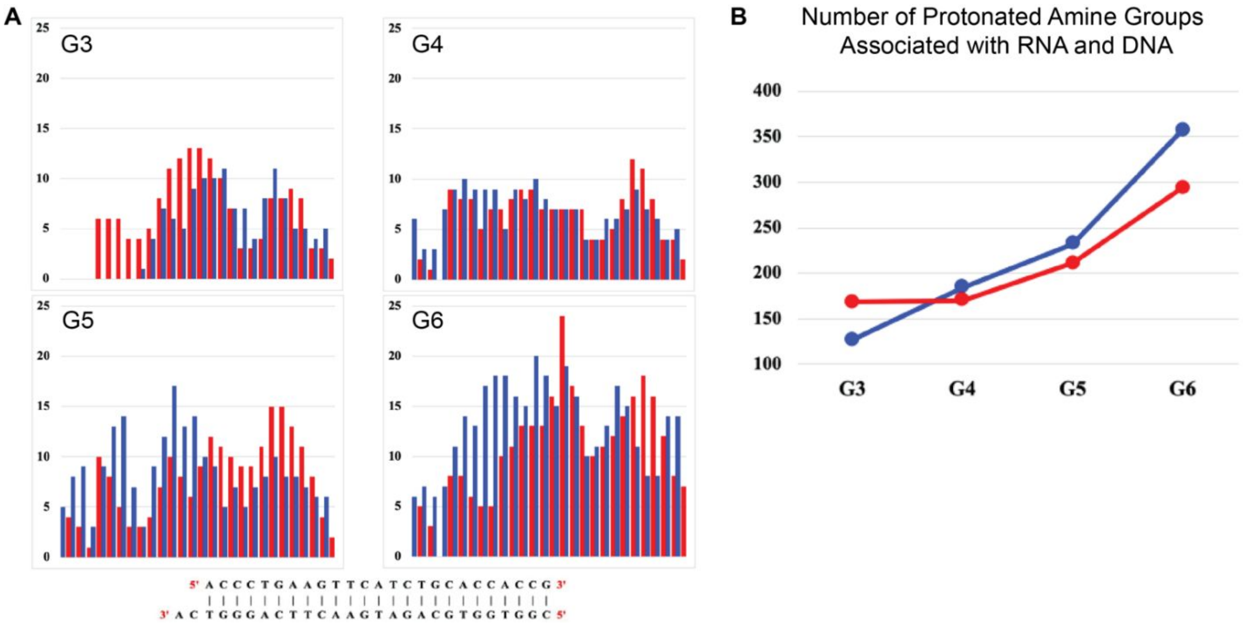

Figure S3. (**A**) Number of protonated amine groups located within 12 Å of DNA (blue) and RNA (red) phosphate groups, shown per base pair. The DNA sequence is used as a reference to represent base pair positions. (**B**) Total number of protonated amine groups associated with DNA (blue) and RNA (red). Note that a single protonated amine group may be electrostatically associated with multiple base pairs.

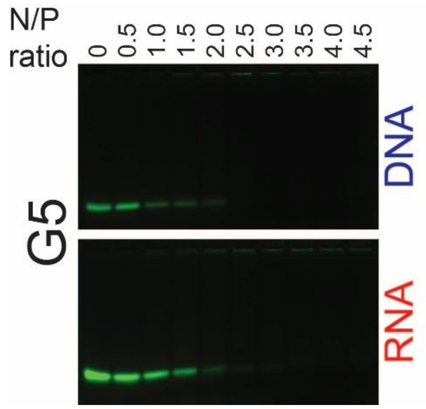

Figure S4. Electromobility shift assays of DNA and RNA duplexes binding with G5 dendrimers.

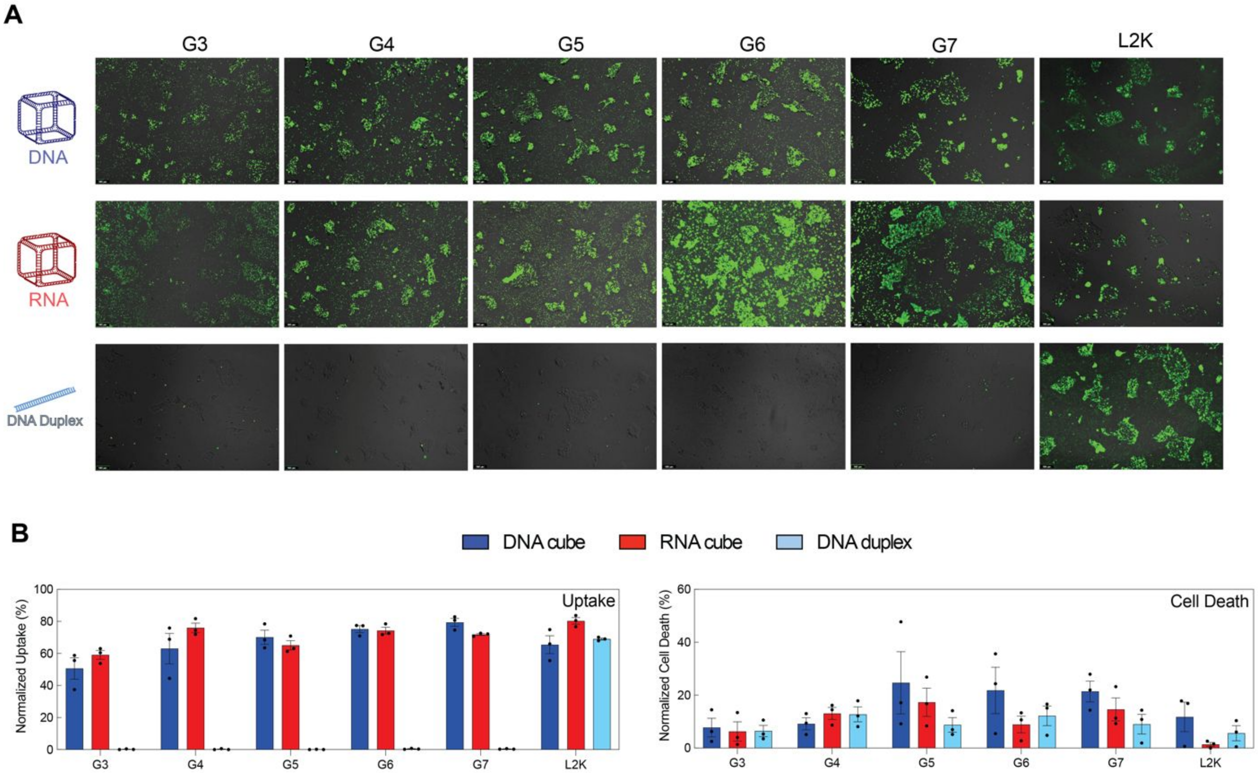

Figure S5. Uptake analysis in HEK-293FT cells treated with Alexa 488-labeled NANPs. (**A**) Merged fluorescence microscopy images showing cellular uptake of DNA cubes, RNA cubes, and DNA duplexes complexed with PAMAM dendrimers of varying generations. Scale bar = 100 µm. (**B**) Flow cytometry analysis using OVERTON gating to assess cellular uptake and viability. Data represent mean ± SEM (N = 3).

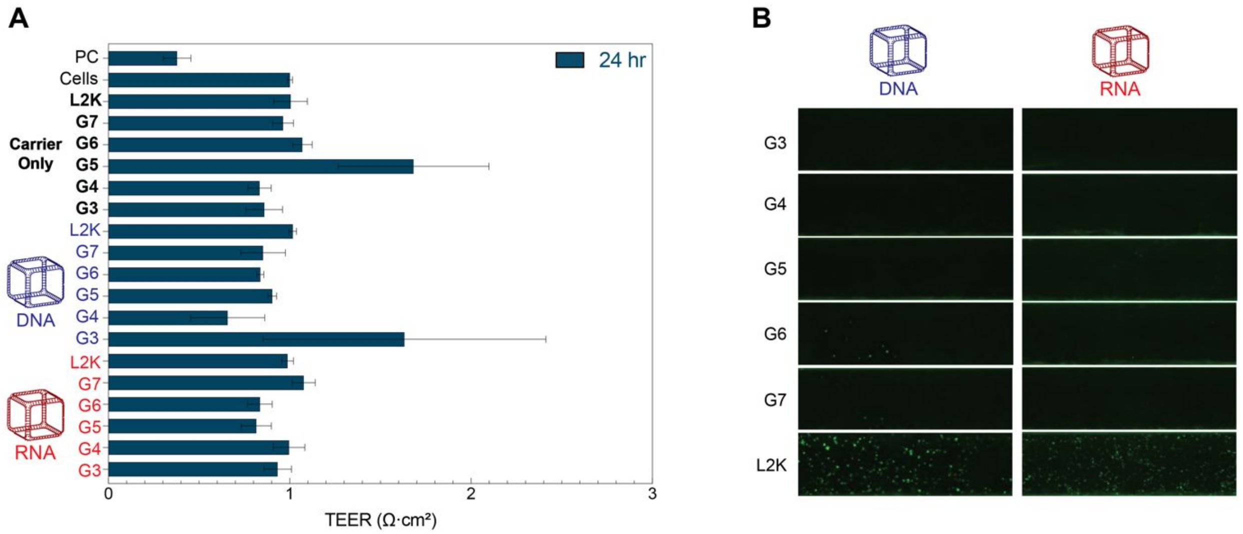

Figure S6. TEER analysis and microscopy images of Caco-2 cultures transfected with dendrimer-nucleic acid complexes. (**A**) Transepithelial electrical resistance (TEER) measurements following treatment with 20 nM DNA or RNA cubes complexed with different generations of dendrimers. Control conditions include vehicles alone, and positive control that disrupts the barrier integrity. TEER values are normalized against t=0, and presented as mean ± SEM (N=3). (**B**) Representative microscopy images of OrganoPlate cultures at 4× magnification showing fluorescent uptake of dendrimer-NANP complexes for 24 hrs. The microscopy images show the uptake of NANPs within the Caco-2 3D tubule, where the uptake increases as the generation of dendrimers increases from G3-G7.

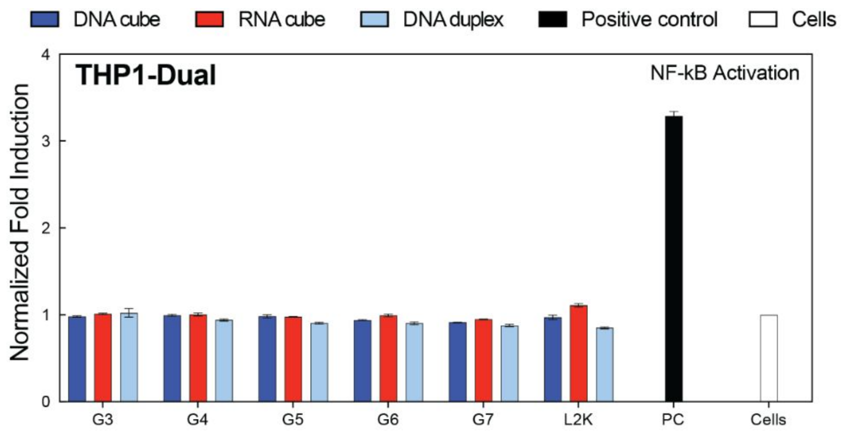

Figure S7. Assessment of NF-kB immune activation in THP1-Dual cells presented as mean ± SEM (N = 3).

SUPPORTING TABLES

	PAMAM dendrimer	Nucleic acids	N/P ratios
	Number of protonated amine groups	Number of phosphate groups	
G3	32	50	0.64
G4	64		1.28
G5	128		2.56
G6	256		5.12
G7	512		10.24

Table S1. The theoretical number of protonated amine groups in PAMAM dendrimers, the number of phosphate groups in nucleic acids, and N/P ratios.

	DNA (kcal/mol)		RNA (kcal/mol)		ΔΔG (DNA-RNA, kcal/mol)	
	PB	GB	PB	GB	PB	GB
G3	-712.8	-288.0	-755.6	-357.8	42.8	69.8
G4	-730.7	-453.9	-832.9	-389.4	102.2	-64.5
G5	-840.7	-471.4	-1485.7	-604.0	645	132.6
G6	-944.2	-776.2	-703.1	-498.6	-241.1	-277.6

Table S2. The results of MM-PB/GBSA. Here, ΔΔG = (binding free energy DNA/dendrimer) − (binding free energy of RNA/dendrimer).
